# Theoretical Study on the Grafting Reaction of Maleimide to Polyethylene in the UV Radiation Cross-Linking Process

**DOI:** 10.3390/polym10091044

**Published:** 2018-09-19

**Authors:** Hui Zhang, Yan Shang, Hong Zhao, Chunyang Li, Xuan Wang, Baozhong Han, Zesheng Li

**Affiliations:** 1 Key Laboratory of Engineering Dielectrics and Its Application of Ministry of Education & College of Chemical and Environmental Engineering, Harbin University of Science and Technology, Harbin 150080, China; shangyan1972@126.com (Y.S.); lichunyang_hust@163.com (C.L.); wangxuan@hrbust.edu.cn (X.W.); 2 Shanghai Qifan Cable Co., Ltd., Shanghai 200008, China; 3 Key Laboratory of Cluster Science of Ministry of Education & School of Chemistry, Beijing Institute of Technology, Beijing 100081, China; zeshengli@bit.edu.cn

**Keywords:** UV radiation cross-linking polyethylene, graft, maleimide, transition

## Abstract

Theoretical investigation of the reaction of graft maleimide to polyethylene in the UV radiation cross-linking process is accomplished at the B3LYP/6-311+G(*d*,*p*) level for high-voltage cable insulation materials. The reaction potential energy surface of the nine reaction channels is identified. The results show that the *N*,*N*′-ethylenedimaleimide can connect two 4-methylheptane molecules and act as the cross-linking agent. The calculated reaction potential barrier of forming 4-methylheptane radical by maleimide is higher than that of maleic anhydride. The study is expected to provide a basis for optimizing the UV radiation cross-linking polyethylene process and development more than 500 kV high-voltage cable insulation materials in practical applications.

## 1. Introduction

With the fast development of new renewable energies, the highly efficient transmission of electricity is becoming more and more important. Offshore wind power and photovoltaic power transmission call for long distance and large capacity high-voltage direct current (HVDC) cable power transmission. This will promote the rapid development of polymeric HVDC cables in the near future. One of the most important issues is the insulation materials. Except for the electrical tree, cross-linked polyethylene (XLPE) insulation for HVDC cable also faces the problem raised by space charge accumulation in the bulk of the insulation. Under high electrical fields, charge carriers and ions in HVDC cable insulation can be trapped by either polymer defects and or polar molecules, forming non-uniform accumulation. Space charge accumulation can affect the operation of the direct current (DC) cable insulation in many ways, such as by distorting local electric field, accelerating material aging, or triggering electrical trees [1,2,3], etc. At present, there are three ways to solve this problem. The first is to manufacture the XLPE insulation cable materials with “chemical purity”, e.g., reducing chemical impurities, such as the antioxidant and cross-linking agent. They may be the origin of the traps for space charge. However, the trap centers in the XLPE include not only the polar group of small molecules, but also the structure defects generated from the polyethylene (PE) molecular chain and its crystallization, such as free volumes and polar groups connected to the chain. The second solution is to introduce nanoparticles to XLPE materials, where the interface between nanoparticles and the polymer matrix may form the deep traps of charge carriers [4,5,6]. However, this strategy has many obstacles in industrial applications, such as blocking of fine mesh during polymer extrusion. The last solution is to graft maleic anhydride (MAH) to the PE chain, which may inhibit space charge accumulation due to the strong polarity [7,8,9]. The alkenyl in MAH ensures grafting with two carbonyl groups as deep traps. But this method has a disadvantage for industrial application, as MAH can vaporize at very high temperature during a real cross-linking process, or generate gel content during the grafting process before cross-linking in the traditional technology. UV radiation technology does not require the high temperatures and may be a good solution for the manufacture of HVDC cable insulation grafted with polar groups [10,11,12,13,14]. In a previous paper, we reported the possible chemical reactions of MAH grafting to the PE chain during UV radiation cross-linking [15]. Some experimental studies have confirmed that maleimide and its derivatives can be used as a modifier for polymer to improve its specific performance, which can also be grafted onto PE and function as a photoinitiator [16,17,18,19,20]. Bismaleimide, which has two ethylene groups and four carbonyl groups, may act as the cross-linking agent and deep traps grafted to the macromolecules. It is known that additional bismaleimide with a dual function used in the system would reduce the chemical impurities of the material.

In this work, 4-methylheptane (Pe) was selected as model molecules of the cross-linkable PE. Two kinds of polar molecules, including butene diacid derivatives and acrylic acid derivatives, were selected to study their excitation energies. The butene diacid derivatives include maleimide (MAM), *N*-methylmaleimide (MMM), *N*-ethylmaleimide (EMM), *N*-carbamoyl maleimide (CMM), *N*-phenylmaleimide (PM), *N*,*N*′-ethylene dimaleimide (EEM), maleic anhydride (MAH), ethyl hydrogen maleate (EH) and methyl hydrogen fumarate (MHF). The acrylic acid derivatives include *trans*-3-chloro acrylic acid (TRCC), allyl chloroacetate (ACA), acrylic acid (AA) and methacrylate (MA). The main materials for preparing XLPE according to the UV radiation cross-linking process include cross-linkable PE, photoinitiator benzophenone (Bp), space charge inhibitor, hindered phenol antioxidant and multi-functional cross-linker triallyl isocyanurate (TAIC). The possible reactions of grafting CMM or EEM to PE, and then further grafting EEM–*g*–Pe to PE by UV radiation have been investigated. What is the reaction between maleimide and PE? How does the reaction proceed? Subsequently, is it difficult for EEM–*g*–Pe to graft to PE? The answers to these questions are not very clear, currently. The mechanism of UV radiation on the chemical reaction of grafting polar molecules to PE will contribute to the optimization of the process of UV radiation cross-linking and promote the development of insulating materials in practical applications.

## 2. Computational Methods

The B3LYP [21,22,23,24] functional with the 6-311+G(*d*,*p*) basis set was used for the equilibrium geometry optimizations and frequency calculations of the studied molecules at the ground state or the triplet state by using the density functional theory (DFT) method [25]. The transition state was identified by having only one imaginary frequency. This level of B3LYP/6-311+G(*d*,*p*) has been proved to be suitable for current research in our previous paper where the calculated results are consistent with the corresponding experimental results [26]. The time-dependent density functional theory (TDDFT) method [27,28] was employed to calculate the excitation energies of two kinds of polar molecules on the basis of the optimized geometries at the B3LYP/6-311+G(*d*,*p*) level. Three lowest excitation states (S_1_, S_2_, and S_3_) of each polar molecule were computed. The natural bond orbital (NBO) method [29] was used to analyze the natural charge population on 9 sites of maleimide. The minimum energy path (MEP) was obtained by intrinsic reaction coordinate (IRC) calculations with a gradient step-size of 0.05 (amu)^1/2^ bohr. The first and second energy derivatives were obtained to calculate the curvature of the reaction path and the generalized vibrational frequencies along the reaction path. All calculations were performed by the GAUSSIAN09 program package [30].

## 3. Results and Discussion

### 3.1. Stationary Point Geometries

The excitation energies (S_1_, S_2_, and S_3_) of two kinds of polar molecules have been calculated at the B3LYP/6-311+G(*d*,*p*) level. The computational results are listed in Table 1 together with the molecular formulas and corresponding abbreviations (ab.). In Table 1, the results show that maleimide derivatives, which have lower electron excitation energy levels than Bp, would be more-easily grafted to the PE molecule by UV radiation without the help of Bp, and could be selected as a new candidate for a highly efficient space charge inhibitor. The excitation energies of EH, MHF, and the acrylic acid derivatives are all higher than that of Bp. This means that they would be grafted to PE by UV radiation with the help of Bp if their energy level of frontier molecular orbitals accords with sensitization conditions. The cross-linking byproduct from Bp and TAIC will affect some electrical properties such as increasing conductivity of the insulation. EEM, which has two ethylene groups, would act as the cross-linking agent and connect two PE molecules when it is excited by UV initiated without the help of Bp and TAIC.

**Figure 1:**
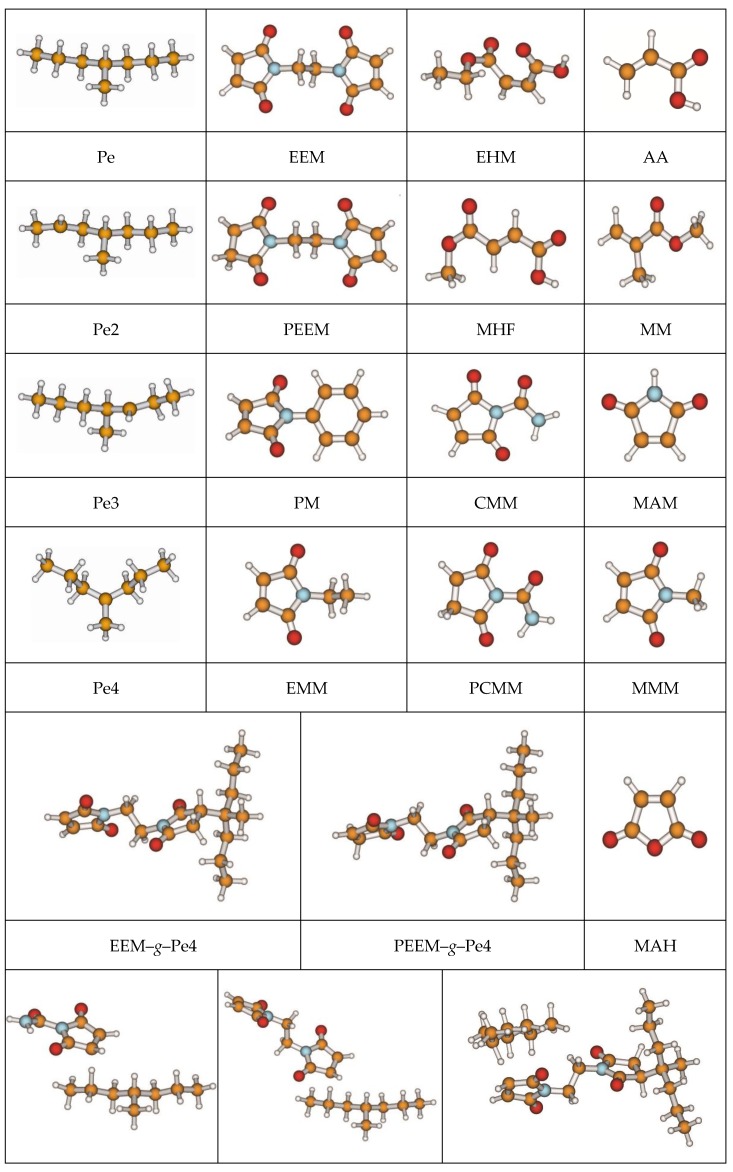

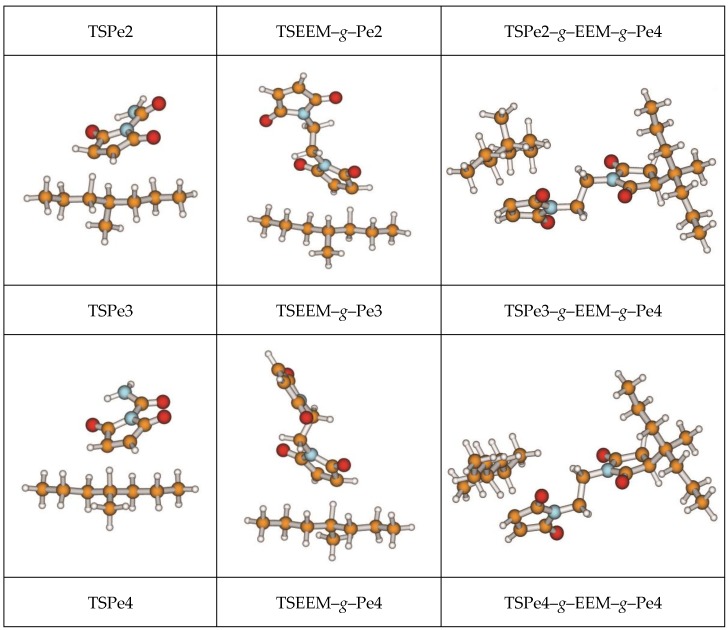
Optimized geometric structures of the studied molecules at the B3LYP/6-311+G(*d*,*p*) level.

In Table 2, hydrogen abstraction reactions for the grafting of maleimides to PE by UV radiation are listed. The optimized geometric structures at the triplet state of the reactants, the transition states involved in the nine ultraviolet light induced reactions, and other stationary points at the ground state are presented in Figure 1. The optimized lengths for the breaking and forming bonds in nine transition states, and the corresponding values for C–H bonds in both the reactant and the product, as well as the imaginary frequency values, are also listed in Table 2. In this paper, R, TS, and P refer to the reactant, the transition state, and the product, respectively.

In Table 2, it can be seen that among the nine studied reactions, the transition state structures at the T_1_ state of the hydrogen abstraction reaction have a common characteristic in that the breaking elongation of the reactant (C–H in model molecule Pe) is less than the bond elongation of the products (C–H in PCMM, PEEM, PEEM–*g*–Pe4 and PPe4–*g*–EEM–*g*–Pe4), indicating that the transition states are closer to the reactant and belong to the “early” transition state. According to Hammond’s postulate [31], this kind reaction should be exothermic.

### 3.2. Frontier MOs and NBO Charge Population

According to our previous study [32], the Si*_x_*O*_y_* nanoclusters have been proved to be efficient in accumulating electrons and protecting the PE chain. Our previous study also showed that the calculated vertical and adiabatic values of the ionization potentials (IPs) and the electron affinities (EAs) of MAH at the B3LYP/6-311+G(*d*,*p*) level are higher than those of the other two polar molecules (MA and AA) [15]. In this work, the calculated IPs and EAs for polar molecules CMM and EEM at the same level are higher than those of MAH. When grafted by CMM or EEM, the polymer would have a much stronger ability to suppress space charge injection and accumulation than if grafted by MAH under divergent electrical field stress in-service XLPE cables. CMM (1.80 eV) and EEM (1.76 eV) have higher EA(*a*) values than MAH (1.72 eV), suggesting that when grafted to PE, CMM and EEM would have much stronger abilities of capturing the hot electron with C=O groups in the insulation materials than MAH. The calculated highest occupied molecular orbital and the lowest unoccupied molecular orbital (HOMO–LUMO) energy gaps, *E*_g_, values of 4.36 and 4.68 eV for CMM and EEM, respectively, are lower than 5.01 eV for MAH, although all are α,β-unsaturated carbonyl compounds. Because there are acylamino groups (–NC=O) in CMM and EEM, the conjugative effect between C=O groups and the N atom in maleimides is larger than that of C=O groups and the O atom in MAH. The electronic density on the C=C double bond relates to the electronegativity of the atom linked to acyl groups: the larger the electronegativity, the stronger the electron-withdrawing capability. Because the electronegativity of the O atom is larger than that of the N atom, the electronic density on the C=C double bond of MAH obviously decreases. Therefore, the reactivity of CMM or EEM to PE would be lower than that of MAH.

The natural charge population on nine sites of MAM, CMM, and EEM at S_0_, S_1_, and T_1_ states is given in Table 3. The maleimide molecules at the excited triplet state, T_1_, have double radicals which can reconstruct π bonds. The natural charge density on C1 and C2 of the CMM molecule at the excited triplet state, T_1_, is higher than that of EEM. As a result, C1 and C2 in EEM show higher reactivity on the excited triplet state than in CMM. The reaction potential barrier heights of EEM would be lower than those of CMM.

### 3.3. Energetics

During the thermo-crosslinking process of PE, dicumyl peroxide takes a homolytic reaction and forms radicals [33], which initiate a hydrogen abstraction reaction with the PE chain to form PE radicals [26]. In the process of grafting MAH to PE by UV radiation, the benzophenone Bp is excited to the excited singlet state at first, and then to the excited triplet state through the intersystem crossing (ISC). Bp at the excited triplet state can sensitize MAH from the ground state to the excited triplet state, and return to the ground state. MAH at excited triplet state abstracts the hydrogen from the PE to form PE and MAH radicals. These PE radicals can react quickly with each other to produce a cross-linked network XLPE, through which mechanical properties and heat resistance are significantly enhanced, and MAH can also be grafted to the PE chain [26]. The radical reactions are induced by Bp, and the cross-linking rate is graded by TAIC [34]. Compared with the process of thermal initiated grafting with peroxide as initiator, photoinitiated grafting provides higher grafting efficiency [10]. In Table 4, at the B3LYP/6-311+G(*d*,*p*) level, the calculated reaction enthalpies at 298 K (Δ$H_{298}^{0}$) and the potential barrier heights (Δ*E*^TS^) with zero-point energy (ZPE) corrections at T_1_ state are presented; the relative bond dissociation energies ($D_{298}^{0}$) are also provided. Bond dissociation energies have good correlations with the corresponding reaction potential barrier heights. The calculated relative energy margin between S_0_ and T_1_ states (Δ*E*^T1-S0^) of acetophenone was 74.92 kcal/mol at the QCISD(T)/B3LYP level in our previous work [35], which is consistent with the experimental value of 73.74 kcal/mol [36]. Here, we aim at investigating the possible reactions of maleimides with Pe by UV radiation. Which can easily graft to PE chain, CMM or EEM? Can EEM act as the cross-linking agent and connect two Pe molecules?

During the UV radiation cross-linking PE process, the calculated reaction potential barrier of forming the Pe2 radical by MAH is 0.10 eV at the T_1_ state at the B3LYP/6-311+G(*d*,*p*) level [15]. The calculated reaction potential barrier of forming the Pe2 radical by EEM is 0.26 eV at the T_1_ state at the same level in this work, which is higher than that of MAH. This is consistent with a qualitative assessment based on the electronic density analysis above. Therefore, the electronic density on the C=C double bond in MAH is smaller than that of maleimide (EEM and CMM), leading to the more facile MAH radical with the lower energy barriers (0.10 eV) [15]. For the nine forming radical reactions with Pe in Table 4 at the T_1_ state, all are exothermic, consistent with Hammond’s postulate [31]. The reaction potential barrier of forming the Pe4 radical by EEM (0.19 eV) is the lowest among the nine reaction channels and the reaction enthalpy (Δ$H_{298}^{0}$ = −0.47 eV) is also the lowest. As a result, the reaction channel REEM–*g*–Pe4 is more thermodynamically and kinetically favorable than others. Moreover, the reaction potential barrier of the hydrogen abstraction reaction TSPe4–*g*–EEM–*g*–Pe4 by PEEM–*g*–Pe4 from Pe4 is 0.20 eV, and the reaction enthalpy is −0.46 eV, meaning that the reaction channel of RPe4–*g*–EEM–*g*–Pe4 has kinetic and thermodynamic superiority. That is to say, EEM can act as the cross-linking agent and connect two Pe molecules. This has been mentioned in experimental investigation [20]. In Table 4, it can be seen that the reaction potential barrier of the grafting of CMM to Pe is higher than that with EEM; this is in line with the natural charge population results discussed above. Thus, grafting of EEM to Pe is easier than CMM. Grafting the maleimide to the PE molecule chain can introduce uniformly and densely distributed deep traps in the PE insulation material; the electrons emitted from the cathode under a high electrical field would be trapped in the interface and form a lattice-like charge point distribution, causing the Coulomb force field to reject further injection of the electrons from cathode. If the cross-linking agent EEM can be used, it would not only be excited without the help of Bp and TAIC in the UV radiation cross-linking PE production process, but also reduce the chemical impurities in the PE insulation material. This insulation material system, with four carbonyl groups as deep traps, would have better space charge suppression characteristics. If gel does not form during EEM grafting to PE in the UV radiation cross-linking process, CMM–*g*–Pe and Pe–*g*–EEM–*g*–Pe will form a “space lattice”, and thereby inhibit space charge accumulation effectively. Further experimental and theoretical studies are needed to optimize the UV radiation cross-linking PE process and develop over 500 kV high-voltage cable insulation materials in practical applications.

## 4. Conclusions

A systematically theoretical study on the grafting reaction mechanisms of maleimide to polyethylene by UV radiation has been carried out at the B3LYP/6-311+G(*d*,*p*) level. *N*,*N*′-ethylenedimaleimide EEM with two ethylene groups and four carbonyl groups can be foremost elucidated to act as a cross-linking agent to provide a charge trap function. The system has a high degree of simplicity and less reaction byproduct to affect electrical properties. We expect excellent electrical properties of prepared insulation material. EEA can connect two Pe molecules, and the grafting of EEM to PE is easier than that of CMM. The potential energy surface information of the nine reaction channels and the excitation cross-linking agent would assist the rational design of space charge inhibitors and the optimization of the UV radiation cross-linking process. Here we propose using the excitation energies and the reaction potential barrier heights as guiding criteria for identifying novel high-efficiency cross-linking agents with charge trap functions, which opens up the vast library of maleimide for potential candidates for the design of HVDC power cable insulation material in real applications.

## Figures and Tables

**Table 1:** Calculated excitation energies (in eV) at the excited singlet state (S_1_, S_2_, and S_3_) of the studied polar molecules at the B3LYP/6-311+G(*d*,*p*) level.

ab.	Molecular Formula	S*_n_*	Excitation Energy	ab.	Molecular Formula	S*_n_*	Excitation Energy
BP	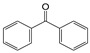	1	3.5991	MAH	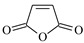	1	3.6485
		2	4.5270			2	4.3668
		3	4.6060			3	5.3341
CMM	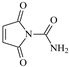	1	3.2558	EH	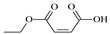	1	4.5740
		2	3.7502			2	4.7433
		3	3.9501			3	5.1899
MAM	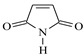	1	3.4273	MHF	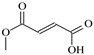	1	3.8208
		2	4.2021			2	4.2214
		3	4.5092			3	5.0101
EEM	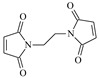	1	3.4650	TRCC	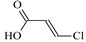	1	4.6060
		2	3.4650			2	5.7016
		3	3.9339			3	6.0774
MMM	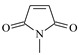	1	3.4672	ACA	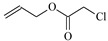	1	5.1686
		2	4.0443			2	5.9846
		3	4.1825			3	6.1150
EMM	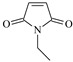	1	3.4604	AA	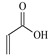	1	4.6843
		2	4.0070			2	6.3785
		3	4.1725			3	6.6245
PM	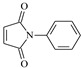	1	3.1134	MM	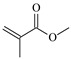	1	4.8253
		2	3.4628			2	5.8684
		3	3.5347			3	6.4778

**Table 2:** Hydrogen abstraction reaction equations for the grafting of maleimides to PE by UV radiation at the triplet state, the lengths of the reaction-involved C–H bonds in reactants, transition states (the breaking bond/the forming bond, *b*/*f*), products (in angstrom) and the imaginary frequency values (in cm^−1^) of the transition states.

Reaction Equation	Reactant	*b*/*f*	Product	Frequency
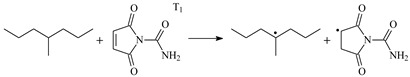	1.100	1.280/1.479	1.098	964 *i*
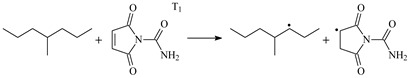	1.098	1.302/1.438	1.098	1199 *i*
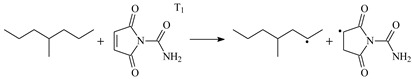	1.097	1.303/1.428	1.098	1185 *i*
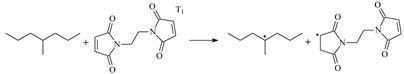	1.100	1.285/1.469	1.097	1080 *i*
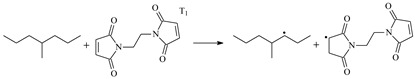	1.098	1.305/1.435	1.097	1288 *i*
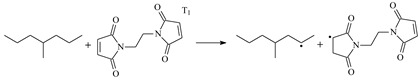	1.097	1.305/1.424	1.097	1280 *i*
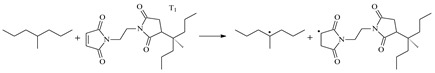	1.100	1.289/1.465	1.097	1118 *i*
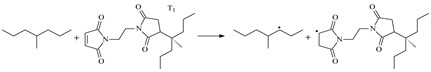	1.098	1.308/1.433	1.097	1307 *i*
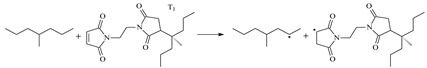	1.097	1.300/1.429	1.097	1257 *i*

**Table 3:** Natural charge population of maleimide at S_0_, S_1_, and T_1_ states.

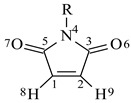			Natural Charge Population								
			C1	C2	C3	N4	C5	O6	O7	H8	H9
R=	H	S_0_	−0.226	−0.226	0.652	−0.674	0.652	−0.534	−0.534	0.232	0.232
		S_1_	−0.207	−0.207	0.620	−0.660	0.620	−0.534	−0.534	0.238	0.238
		T_1_	−0.298	−0.297	0.538	−0.614	0.537	−0.382	−0.382	0.237	0.237
R=	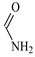	S_0_	−0.225	−0.207	0.672	−0.594	0.673	−0.473	−0.562	0.236	0.236
		S_1_	−0.227	−0.166	0.617	−0.571	0.661	−0.432	−0.538	0.241	0.242
		T_1_	−0.278	−0.297	0.525	−0.530	0.592	−0.264	−0.610	0.231	0.235
R=	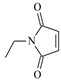	S_0_	−0.222	−0.221	0.666	−0.543	0.668	−0.539	−0.538	0.232	0.232
			−0.222	−0.221	0.666	−0.543	0.668	−0.539	−0.538	0.232	0.232
		S_1_	−0.215	−0.194	0.626	−0.532	0.647	-0.549	−0.527	0.237	0.239
			−0.222	−0.221	0.665	−0.543	0.668	-0.537	−0.539	0.232	0.232
		T_1_	−0.142	−0.142	0.549	−0.382	0.552	-0.590	−0.587	0.217	0.217
			−0.220	−0.220	0.666	−0.539	0.669	-0.536	−0.533	0.233	0.233

**Table 4:** The reaction enthalpies at 298 K (Δ$H_{298}^{0}$), the potential barrier heights TSs (Δ*E*^TS^) with zero-point energy (ZPE) corrections at the T_1_ state at the B3LYP/6-311+G(*d*,*p*) level, and the bond dissociation energies of C–H bonds in the reactants (all in eV).

Reaction Equation	B3LYP/6-311+G(*d*,*p*)		
	Δ*E*^TS^+ZPE	Δ$H_{298}^{0}$	$D_{298}^{0}$
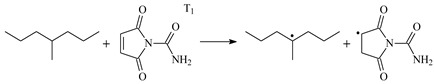	0.41	−0.18	3.91
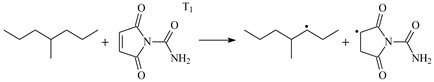	0.53	−0.06	4.04
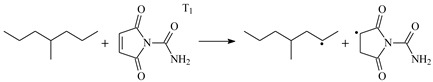	0.49	−0.09	4.01
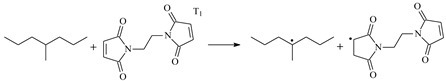	0.19	−0.47	3.91
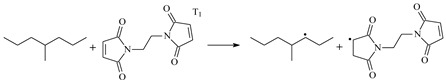	0.30	−0.35	4.04
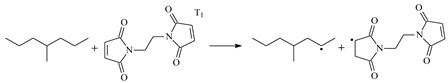	0.26	−0.38	4.01
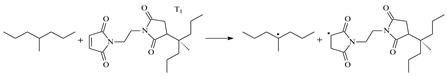	0.20	−0.46	3.91
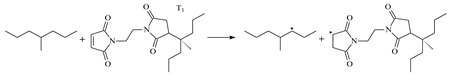	0.32	−0.34	4.04
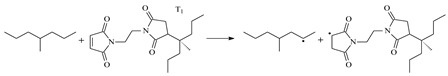	0.25	−0.37	4.01
